# Platinum (IV)-fatty acid conjugates overcome inherently and acquired Cisplatin resistant cancer cell lines: an in-vitro study

**DOI:** 10.1186/s12885-016-2182-8

**Published:** 2016-02-23

**Authors:** Einav Ratzon, Yousef Najajreh, Rami Salem, Hazem Khamaisie, Martin Ruthardt, Jamal Mahajna

**Affiliations:** Cancer Drug Discovery Program, Migal, Galilee Research Institute, P.O. Box 831, Kiryat Shmona, 11016 Israel; Anticancer Drugs Research Lab, Faculty of Pharmacy, Al-Quds University, P.O. Box 20002, Jerusalem, Abu-Dies, Palestinian Authority; Medizinische Klinik II/Abtl. Hämatologie, Klinikum der Johann Wolfgang Goethe Universität, Theodor-Stern Kai 7, 60590 Frankfurt, Germany; The Department of Nutritional Sciences, Tel Hai College, Kiryat Shmona, Israel

**Keywords:** Platinum (IV), Cisplatin, Ovarian cancer, Resistance, Copper transporter (Ctr1)

## Abstract

**Background:**

Platinum-based drugs are used as cancer chemotherapeutics for the last 40 years. However, drug resistance and nephrotoxicity are the major limitations of the use of platinum-based compounds in cancer therapy. Platinum (IV) complexes are believed to act as platinum prodrugs and are able to overcome some of platinum (II) limitations.

**Methods:**

A number of previously sensitized platinum (IV) complexes were evaluated for their anti-cancer activity by monitoring ability to affect proliferation, clonigenicity and apoptosis induction of Cisplatin sensitive and resistant cancer cells. In addition, the uptake of Cisplatin and the platinum (IV) derivatives to Cisplatin sensitive and resistant cancer cells was monitored.

**Results:**

The bis-octanoatoplatinum (IV) complex (RJY13), a Cisplatin derivative with octanoate as axial ligand, exhibited strong anti-proliferative effect on the Cisplatin resistant and sensitive ovarian cells, A2780cisR and A2780, respectively. Moreover, RJY13 exhibited good activity in inhibiting clonigenicity of both cells. Anti-proliferative activity of RJY13 was mediated by induction of apoptosis. Interestingly, a bis-lauratopaltinum (IV) complex (RJY6) was highly potent in inhibiting clonigenicity of both Cisplatin sensitive and Cisplatin resistant cells, however, exhibited reduced activity in assays that utilize cells growing in two dimensional (2D) conditions. The uptake of Cisplatin was reduced by 30 % in A2780 in which the copper transporter-1 (Ctr1) was silenced. Moreover, uptake of RJY6 was marginally dependent on Ctr1, while uptake of RJY13 was Ctr1-independent.

**Conclusions:**

Our data demonstrated the potential of platinum (IV) prodrugs in overcoming acquired and inherited drug resistance in cancer cell lines. Moreover, our data demonstrated that the uptake of Cisplatin is partially dependent on Ctr1 transporter, while uptake of RJY6 is marginally dependent on Ctr1 and RJY13 is Ctr1-independent. In addition, our data illustrated the therapeutic potential of platinum (IV) prodrugs in cancer therapy.

## Background

cis-Diamminedichloroplatinum (II) [*cis*-[PtCl_2_(NH_3_)_2_], CDDP, Cisplatin] known for decades as Peyrone’s salt, was first synthesized by M. Peyrone in 1845. Its cytotoxic activity was reported in 1964 by Rosenberg [1], and its anti-cancer activity in 1979. Cisplatin is routinely employed for the treatment of testicular and ovarian cancers and is being increasingly used against cervical, bladder, and head/neck tumors. The mechanism of action of Cisplatin is based on the intrastrand cross-linking of the *cis*-Pt(NH_3_)_2_ unit to cellular DNA at two neighboring guanine bases [2] and the consequent induction of cellular apoptosis. Nevertheless, its full clinical utility is limited due to some adverse side effects.

Primary and acquired drug resistance is a major limitation of the platinum compounds use as an anti-cancer therapy [3, 4]. The molecular mechanisms that underline this chemo resistance are largely unknown. Possible mechanisms include decreased platinum accumulation, elevated drug inactivation by metallothionine and glutathione, and enhanced DNA repair activity [2, 5]. Moreover, acquired and inherited resistance of cancer cells were reported to be also mediated by altered molecular mechanisms and activated signaling pathways, such as the protein kinase B/mammalian target of rapamycin (Akt/mTOR) which is also implicated in Cisplatin resistance in human ovarian cancer cells [6].

**Fig. 1:**
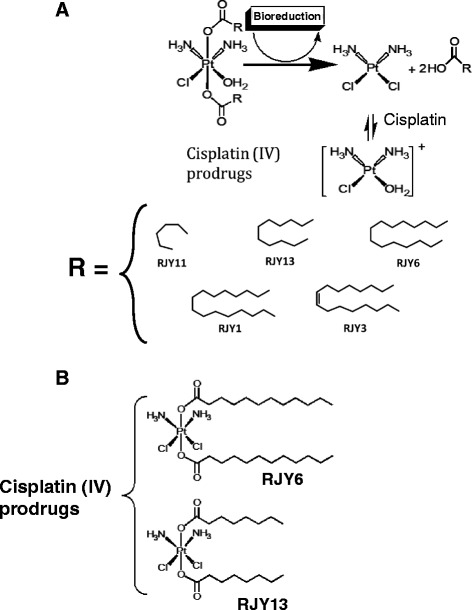
Chemical structure of Cisplatin (IV) prodrugs and cellular bio-reduction. **a**. Bio-reduction of Cisplatin (IV) prodrugs to give rise to the biologically active Cisplatin moiety and the different fatty acid ligands (R). **b**. Chemical structure of the two Cisplatin (IV) prodrugs; RJY6 and RJY13

In an attempt to overcome the above mentioned shortages, two platinum compounds were introduced to the clinic; Carboplatin and Oxaliplatin [7, 8]. Carboplatin, though much less potent than Cisplatin, have shown fewer adverse effects. Nonetheless, the drug showed cross-resistance with Cisplatin, while Oxaliplatin did not [9, 10]. Moreover, a third generation orally available lipophilic platinum, Satraplatin, demonstrated promising antitumor activity in multiple settings with a better toxicity profile than Cisplatin [11, 12]. However, it has recently been abandoned in phase III clinical trials for the treatment of hormone-refractory prostate cancer [13]. It is well-established that platinum (II) adverse effects are caused mainly by its ability to non-selectively bind to macromolecules, leading to reduced bioavailability and increased toxic side effects. Platinum (IV) complexes, on the other hand, have enormous potential as anticancer agents in terms of both high activity and low toxicity. These potential advantages of Platinum (IV) complexes, which expected to remain in higher oxidation state in the bloodstream, are derived from their lower reactivity towards macromolecules, which enables to diminish the loss of active drug and lower the incidence of unwanted side reactions that lead to toxic side effects. The octahedral platinum (IV) geometry makes these compounds far more kinetically inert towards ligand exchange reactions and less prone to substitution reactions in the physiological media, thus making such compounds of high interest in avoiding the adverse toxic effects seen with platinum (II). In addition, once entered the cell, platinum (IV) is bio- converted to the corresponding platinum (II) species by expelling the axial ligands (Fig. 1). Together, the above mentioned observations laid down the bases for the rational that Platinum (IV) compounds have the potential of acting as prodrugs for their counter platinum (II) active forms (Fig. 1) [14]. However, the first such chemotherapeutic agent, namely satraplatin, failed to receive approval. Moreover, others also questioned the above prodrug rational and pointed toward the ability of paltinum (IV) prodrug to be reduced extracellularly [15, 16].

Recently, a series of complexes of the general formula *cis*, *cis*, *trans*-[diamminedichloro-bis-carboxylatoplatinum(IV)], where the carboxylate ranges between heptanoate, octanoate, decanoate ,laurate, myristate, palmitoate , stearate , and oleateandelaidate, were prepared (Fig. 1), characterized, and their anti-proliferative and anti-clonigenicity properties against cancer cells were evaluated. Our results showed that complexes encompassing saturated fatty acid derivatives were generally more potent than the unsaturated ones.

Two promising platinum (IV) prodrugs, RJY6 and RJY13 were selected for the evaluation of their anti-cancer activity against several cancer cell lines including ovarian and colon cancer cells. Here, we report that while RJY13 was highly potent in inhibiting proliferation and clonigenicity of both Cisplatin sensitive and Cisplatin resistant cancer cells, RJY6 was highly active in the clonigenicity assay but exhibited reduced activity in assays that utilize cells growing in 2D condition (plastic). Moreover, uptake of the two platinum (IV) prodrugs was largely Ctr1-independent which accounts, in part, for the enhanced activity against Cisplatin resistant cancer cells.

## Methods

### Materials

Cisplatin, Carboplatin and Oxaliplatin were purchased from Sigma. Stock solutions were 25–50 mM in phosphate-buffered saline (PBS) and dilution were made with PBS to reach the appropriate concentrations in the different assays. RJYs were synthesized at the laboratory of Anticancer Drugs Research Lab, Faculty of Pharmacy, Al-Quds University, Jerusalem, Abu-Dies, Palestine and dissolve in dimethyl sulfoxide (DMSO) (25-50 mM) and diluted to obtain final DMSO solutions of 0.1–0.5 % in the different assays.

Synthesized Platinum (IV) prodrugs were subjected to proton nuclear magnetic resonance (^1^H-NMR) and ^195^Pt-NMR spectroscopy using Varian Unity Inova 500 MHz spectrometer equipped with a 5-mm switchable and data were processed using the VNMR software. Moreover, infrared spectra were obtained from a KBr matrix (4000–400 cm-1) using a PerkinElmer Precisely, Spectrum 100, fourier transform infrared spectroscopy (FT-IR) spectrometer. Furthermore, to all synthesized prodrugs an Electrospray ionization mass spectrometry (ESIMS) was performed using a ThermoQuest Finnigan LCQ-Duo in the positive ion mode (Najajreh et al., in preparation).

Obtained data for the compound RJY6 (Cis, cis, trans-[diamminedichloro-bis-lauratoplatinum(IV)]) were as follow: yellowish solid product with yield of 35 %, ^195^Pt-NMR: (DMSO-d6, δ ppm) = 1204.25 and FT-IR (KBr) (cm-1): 3403(N-H), 1560 (C = O), 540(Pt-O). Obtained data for the compound RJY13 (Cis, cis, trans-[diamminedichloro-bis-octanoatoplatinum (IV)]): were as follow: yellowish solid product with of 28 %, ^195^Pt-NMR: (DMSO-d6, δ ppm) = 1203.40 and FT-IR (KBr) (cm-1): 3125 (N-H), 1580 (C = O), 527 (Pt-O).

### Cells and cancer cell lines

Colon cancer (HT29), prostate cancer (PC3), Cisplatin sensitive ovarian cancer (2780), and Cisplatin resistant ovarian cancer cell lines (A780cisR) were obtained from ATCC (ATCC, USA). Cells were grown in Roswell Park Memorial Institute (RPMI) 1640 (Sigma, Rehovot, Israel) containing 2 mM L-glutamine, 10 % fetal bovine serum (FBS) , 100 IU/ml penicillin, and 100 μg/ml streptomycin (PenStrep). The human embryonic kidney cell line 293A (HEK293A), human embryonic kidney cell line 293 T (HEK293T) and human foreskin fibroblast cells (HFF) were maintained in Dulbecco’s Modified Eagle’s Medium (DMEM) medium (Sigma, Rehovot, Israel) supplemented with 10 % fetal calf serum (FCS), 2 mM L-glutamine, 1 mM sodium pyruvate, and 1 % PenStrep (Biological Industries, Israel). All cell lines were grown at 37 °C in a humidified atmosphere with 5 % CO_2_.

### Cell proliferation assay

(2,3-bis-(2-methoxy-4-nitro-5-sulfophenyl)-2H-tetrazolium-5-carboxanilide) (XTT) assay was used as previously described [17] to evaluate the cytotoxicity of the different Cisplatin derivatives. Briefly, cells (1.5 × 10^4^) were plated in RPMI 1640 medium using 96-well plates for 24 h, and then treated for an additional 72 h with the different Cisplatin derivatives. A total of 50 μl of XTT solution were added to each well and incubated for 3 h at 37 °C. The optical density was measured by a multi-well plate spectrophotometer at 450 nanometers with a references’ wavelength of 630 nm. The concentrations inhibiting cell proliferation by 50 % (IC_50_s) of all tested derivatives were calculated. The experiment was performed in triplicate, and standard deviations were also calculated.

### Apoptosis assay

To monitor apoptosis potential of Cisplatin derivatives we followed cleavage of poly ADP ribose polymerase (PARP) protein [18]. Briefly, cells (2 × 10^5^ cells/ml) were treated with Cisplatin derivatives for the indicated time. Cells were collected, washed once with cold PBS, and lysed in buffer [10 mMTris-HCl (pH 7.4), 100 mMNaCl, 1 mM EDTA, 1 mM EGTA, 1 mM NaF, 20 mM Na4P2O7, 2 mM Na3VO4, 1 % Triton x-100, 10 % Glycerol, 0.1 % sodium dodecyl sulfate (SDS), 0.5 % deoxycholate, 1 mM phenylmethylsulfonyl fluoride (PMSF), for 30 min at 4 °C. Cell lysate supernatants (40 μg protein/each) were resolved on 8 % SDS-polyacrylamide gel electrophoresis, transferred to nitrocellulose membranes, and analyzed by immune-blotting with an anti-cleaved PARP antibody (Cell signaling technology, USA ). Anti α-tubulin antibody (Santa Cruz Co., CA, USA) was used as a loading control.

### PathScan cleaved PARP (Asp214) sandwich enzyme-linked immunosorbent assay (ELISA)

Cell lysates prepared from treated ovarian cancer cells (30 h) were used for quantitative measurement of cleaved PARP according to manufacturer’s instructions (Cell signaling technology, USA). In our experiments, 20 μg of total lysate proteins from each sample were utilized.

### Clonigenicity assay

Clonigenicity assay was performed as previously describe [19]. Briefly, cells (1 × 10^4^) in 1 ml RPMI 10 % FBS medium were diluted in 1 ml of 0.6 % agar to give a final agar concentration of 0.3 % agar. The cells-agar mixture was poured over a hardened agar base in wells of 12-well plates and allowed to solidify. Once the top layer solidified, 1 ml of medium containing different treatments was placed on top to keep the agar moist. The cells were grown at 37 °C in a 5 % CO_2_ humidified atmosphere until colonies were visible (2 weeks). The plates were stained for 4 h with 5 mg/ml 3-(4,5-dimethylthiazol-2-yl)-2,5-diphenyltetrazolium bromide (MTT), and the dye was extracted with 1 ml solubilization buffer (20 % SDS, 50 % N,N-dimethyl-formamide, 25 mM HCL) for 24 h. The optical density was measured at 570 nm wavelength with a reference wavelength of 630 nm.

### Inductively coupled plasma mass spectrometry (ICP-MS)

To monitor the uptake potential of Cisplatin and Cisplatin (IV) prodrugs into A2780, A2780cisR, and A2780 Ctr1^−^ cell lines, experiment was carried out as previously described [20]. Briefly, cells were plated at 5×10^5^ cells/ml, and on the following day compounds RJY6, RJY13 (10 microM), and Cisplatin (50 microM) were added for 1 h. Cells were collected, washed four times with cold PBS. The cells were counted, digested, and the amount of platinum in the cells was determined by ICP-MS. The amount of paltinum/cells was calculated.

### Silencing of Ctr1 in A2780 cells

A2780 cells were transfected with three different shRNA Ctr1 constructs (Sigma-Aldrich, Rehovot, Israel) according to manufacturer instructions. Briefly, Ctr1 shRNA plasmid DNA, pcMV-dR8.2 dvpr and pcMV-VSVG were co-transfected into HEK293T cells (1.5×10^5^cells\ml) using Fugene 6 (Roche Applied Science, Penzberg, Germany) according to manufacturer instructions. The supernatant of the infected cells was collected 48 h post transfection and used to infect A2780 cells, after which Puromycin resistant clones were selected. Levels of Ctr1 were determined in parental and Puromycin resistant A2780 clones to calculate percentage of silencing.

### Statistical analysis

Statistical analysis was performed using Student’s t-test, with significant values set at *P < 0.05 or **P < 0.005.

## Results

### Evaluation the anti-proliferative effects of Cisplatin (IV) prodrugs on ovarian cancer cell lines

Previously, a number of Cisplatin (IV) prodrugs carrying fatty acid ligands were synthesized (Najajreh et al., in preparation). In this report, we evaluated the activity of Cisplatin (IV)-Fatty acid conjugates against A2780 and A2780cisR, ovarian cancer cells that are sensitive and resistant to Cisplatin, respectively (Fig. 1). The anti-proliferative effect of Cisplatin (IV) fatty acid conjugates, in comparison to Cisplatin and Oxaliplatin, are summarized in Table 1.

**Table 1:** Anti-proliferative activity of Cisplatin (IV) prodrugs. Anti-proliferative activity was determined according to Materials and Methods. IC_50_s values of the different derivatives are in microM. Data shown are of representative experiment with CV below 15 % in all samples. Experiment was repeated three times with comparable outcome

	IC50 (μM)		
Compound	A2780	A2780cisR	K562
Cisplatin	1.3	19.2	5.2
Oxaliplatin	12.7	15.5	3.2
Carboplatin	16.8	109	ND
RJY1	>20	>20	34
RJY2	15.3	6	3.8
RJY3	>20	15	9.4
RJY4	20	7	3.9
RJY5	>20	>20	>20
RJY6	0.7	3.3	1.3
RJY9	>20	>20	>20
RJY10	19.2	>20	>20
RJY11	>20	>20	>20
RJY13	0.08	0.57	0.7
RJY18	14	2.1	0.85
RJY19	12.7	10	9.3

Data presented in Table 1 illustrated that the two ovarian cancer cell lines showed varying sensitivity to Cisplatin, with IC_50_ of 1.3 and 19.2 microM against A2780 and A2780cisR, respectively. Similarly, Cisplatin (IV) fatty acid conjugates exhibited variable potency against the two ovarian cancer cell lines tested (Table 1). The IC_50_ values of the two active derivatives, RJY6 and RJY13, were 0.7 and 0.08 microM, respectively, against the sensitive ovarian cancer cells and 3.3 and 0.57 microM, respectively, against the A2780cisR resistant ovarian cancer cells. Thus, RJY13 exhibited increased potency, ranged from 16 to 33 fold against A2780 and A2780cisR, respectively. However, RJY6 exhibited a moderate increase in potency of about 5-6 fold against the two ovarian cancer cell lines. Similarly, the platinum (IV) prodrugs also exhibited enhanced activity against K562; chronic myelogenous leukemia (CML) cell lines (Table 1) and the two active platinum (IV) prodrugs, RJY6 and RJY13 exhibited enhanced anti-CML by 4 and 7 folds, respectively, compared to Cisplatin.

### Clonigenicity inhibition by Cisplatin (IV) prodrugs

**Fig. 2:**
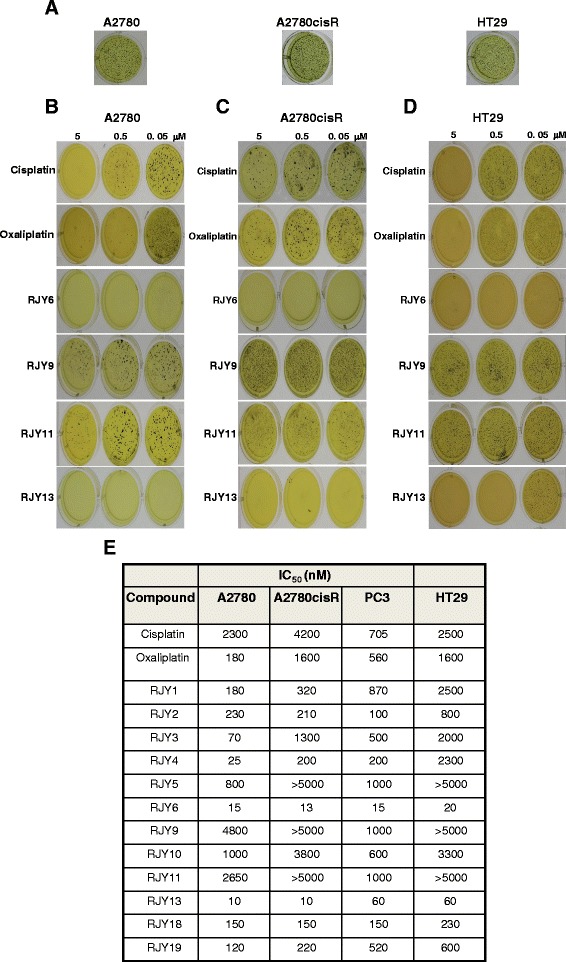
Clonigenicity inhibition of Cisplatin (IV) prodrugs. Cancer cells A2780 (**a**, **b**), A2780cisR (**a**, **c**) and HT29 (**a**, **d**) were grown on soft agar and treated with 5, 0.5 and 0.05 microM of Cisplatin, Oxaliplatin, RJY6, RJY9, RJY11 and RJY13 or solvent-treated cells (**a**) according to Materials and Methods. **e** IC_50_s values of clonigenicity inhibition of the different derivatives are in nM. Data shown are of representative experiment with coefficient of variation (CV) below 15 % in all samples. Experiment was repeated three times with comparable outcome

Anchorage-independent growth of cells (three dimensional, 3D growth) is a typical characteristic of the tumorigenicity of cancer cells in vitro [21]. Thus, the anti-clonigenicity potential of the Cisplatin (IV) prodrugs was evaluated (Fig. 2). Figure 2 shows a photograph of a representative experiment conducted with A2780, A2780cisR, and HT29 cell lines. Clonigenicity inhibition was observed in Cisplatin treated samples with IC_50_ of 2300 and 4200 nM when A2780 and A2780cisR, respectively, were used. Furthermore, Oxaliplatin exhibited enhanced potency toward the two cell lines compared to Cisplatin, with IC_50_ of 180 and 1600 nM against A2780 and A2780cisR, respectively. Interestingly, our platinum (IV) prodrugs exhibited enhanced activity against both cell lines. Our data showed that RJY1, RJY3, RJY4, RJY6, RJY13, RJY18 and RJY19 exhibited good potency against A2780 cells with IC_50_ of 180, 70, 25, 15, 10, 150, and 120 nM, respectively, an increased potency by 10–200 fold among the different derivatives and in comparison to Cisplatin (Fig. 2e). Moreover, a number of derivatives were also effective against the Cisplatin resistant, A2780cisR, cell line, with comparable activity to that observed against the sensitive cell lines, A2780. Of special interest is the derivatives RJY1, RJY2, RJY6, RJY13, RJY18, and RJY19 that effectively inhibited clonigenicity of the Cisplatin resistant ovarian cell line with IC_50_ of 320, 210, 13, 10, 150, and 220 nM, respectively. The two derivatives, RJY13 and RJY6, were significantly more potent than Cisplatin or Oxaliplatin in inhibiting the clonigenicity of the ovarian cancer cell lines tested (Fig. 2) with IC_50_s values lower by more than 20–30 folds than Cisplatin. Moreover, the Cisplatin (IV) prodrugs, and especially RJY13 and RJY6, exhibited significant activity against the inherently resistant cells such as HT29 and PC3 cell lines (Fig. 2).

Next, we selected RJY6 and RJY13 to evaluate their ability to affect proliferation of non-cancerous cells using the HFF, HEK293A and HEK293T cells. HEK293A is an engineered HEK293 cells carrying human species C adenovirus serotype 5 (Ad5) DNA, while HEK293T is carrying the simian vacuolating virus 40 (SV40) large T-antigen. Cisplatin and other platinum (II) compounds exhibited a moderate degree of selectivity toward cancer cells compared to normal cells, mainly due to altered DNA repair mechanism and status of p53 gene. In agreement with published data, we observed that Cisplatin exhibited some degree of selectivity toward A2780 ovarian cancer cell lines compared to HFF, HEK293A and HEK293T (Table 2). Similarly, carboplatin exhibited some degree of selectivity toward the ovarian cancer cells compared to HEK293 cells. Our two platinum (IV) prodrugs also exhibited selectivity toward ovarian cancer cells. RJY13 was more potent against A2780 compared to HEK293A/T by about 10 fold. Interestingly, RJY6 exhibited better selectivity profile of 16 and 24 fold when comparing potency against A2780 to HEK293T and HEK293A, respectively. Moreover, Cisplatin also exhibited good selectivity when comparing potency between A2780 to HFF cells. However, selectivity of RJY13 and RJY6 was reduced by 30 fold and enhanced by 117 fold, respectively, arguing that RJY6 compound is expected to exhibit reduced toxicity to normal tissues and will exhibit an improved therapeutic window. Interestingly, Cisplatin, Carboplatin and RJY6, but not RJY13 exhibited potent activity against HEK293T compared to HEK293A; probably due to compromise DNA repair in HEK293T resulted from impaired function of p53 in those cells.

**Table 2:** Selectivity Index of Cisplatin and active Cisplatin (IV) prodrugs. The IC_50_s of Cisplatin, RJY6 and RJY13 were determined using A2780, HFF, HEK293A and HEK293T cells. Data shown are of representative of three experiments with CV below 20 % in all samples

	IC50 (μM)			
Compound	A2780	HFF	HEK293T	HEK293A
Cisplatin	1.3	84	3.2	16.3
Carboplatin	16.8	ND	45	105
RJY6	0.7	82	11.8	17.3
RJY13	0.08	2.4	0.5	0.7

### Induction of apoptosis by Cisplatin (IV) prodrugs in cancer cell lines

**Fig. 3:**
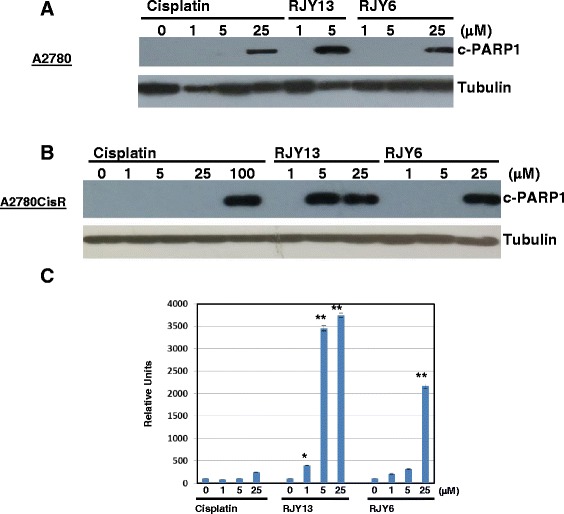
PARP cleavage induced by Cisplatin (IV) prodrugs. A2780cisR (**a**, **c**) and A2780 (**b**) cells were treated with Cisplatin, RJY6 and RJY13 for 30 h as described in Materials and Methods. Quantitative evaluation of cleaved PARP in A2780cisR cells exposed to Cisplatin, RJY6 and RJY13 at (1, 5 and 25 microM) (**c**) were performed as described in Materials and Methods. *P* values; * *P* < 0.05 and **P < 0.005

To investigate whether the effect of the RJY prodrugs is due to apoptosis induction or cell growth suppression, we monitored the ability of the Cisplatin (IV) prodrugs to promote PARP cleavage as a marker of apoptosis induction [18]. Ovarian cancer cells, A2780 and A2780cisR, were treated for 30 h (Fig. 3) with different concentrations of Cisplatin (IV) fatty acid conjugates and results were compared to the effect obtained with Cisplatin. Results shown in Fig. 3 illustrated that treatment with Cisplatin caused very minimal cleavage of PARP in A2780cisR at concentrations below 100 microM (Fig. 3b), while significant PARP cleavage was observed in A2780 cells exposed to 25 microM (Fig. 3a). Interestingly, 5 microM of RJY13 was sufficient to cause a significant PARP cleavage in A2780cisR and A2780 cells (Fig. 3a and b), arguing that RJY13 exhibited enhanced potency against the resistant as well as the sensitive ovarian cancer cells. In contrast, exposer to RJY6 caused PARP cleavage in both A2780cisR and A2780 cells using concentrations above 25 microM, demonstrating enhanced potency to the resistant ovarian cancer cell line in comparison to Cisplatin (Fig. 3a and b). Focusing on Cisplatin resistant ovarian cancer cells (A2780cisR), we performed quantitative measurement of cleaved PARP using Pathscan cleaved PARP (Asp214) sandwich ELISA assay (Cell signaling, USA). Results shown in Fig. 3c demonstrated that Cisplatin was not active in inducing PARP cleavage in A2780cisR cells and only at the highest concentrations used, 25 microM, a 2.4 fold increase in the amount of cleaved PARP was observed (Fig. 3c). In contrast, a significant level of cleaved PARP was observed when A2780cisR were exposed to RJY13. Levels of cleaved PARP were increased by 4, 34 and 37 fold when cells were exposed to 1, 5 and 25 microM of RJY13, respectively (Fig. 3c) compared to untreated sample. Moreover, levels of cleaved PARP were also increased in cells treated with RJY6, but to a lesser extent compared to RJY13 (Fig. 3c). Levels of cleaved PARP were increased by 2, 3 and 21 fold when cells were exposed to 1, 5 and 25 microM of RJY6, respectively (Fig. 3c).

### Involvement of Ctr1 in the uptake of Cisplatin and platinum (IV) prodrugs

**Fig. 4:**
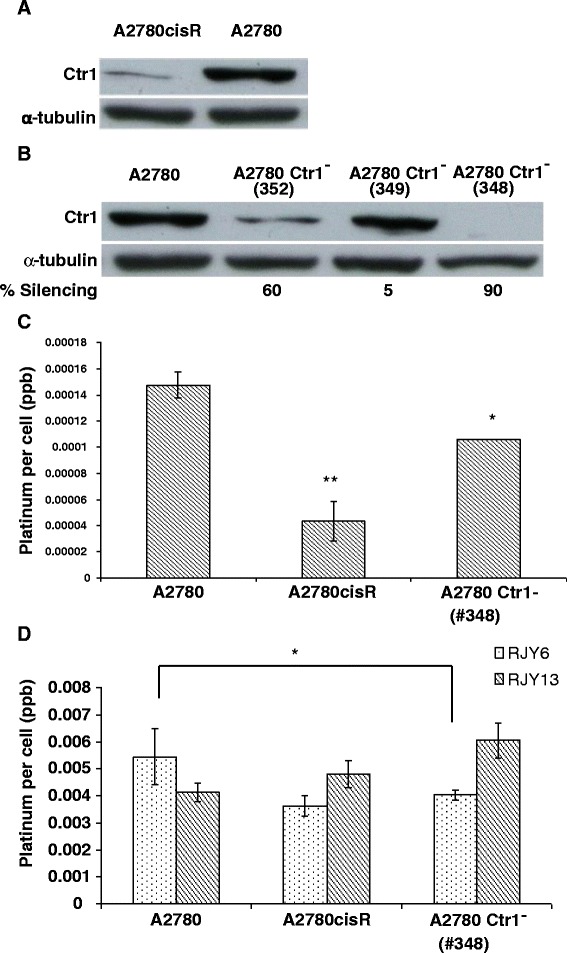
Silencing of Ctr1 and uptake of platinum compounds. **a** Expression of Ctr1 protein was monitored in A2780 and A2780cisR cells. **b** Silencing of Ctr1 gene in A2780 with percentage of decrease in Ctr1 protein levels. **c** Cisplatin uptake into A2780, A2780cisR and A2780 Ctr1^−^ (# 348) cells. **d** Uptake of Cisplatin (IV) derivatives (RJY6 and RJY13) into A2780, A2780cisR and A2780 Ctr1^−^ (# 348) cells. *P* values; * *P* < 0.05 and **P < 0.005

Enhanced expression of hCtr1 was associated with increased accumulation of Cisplatin, arguing for a role of Ctr1 in mediating Cisplatin uptake [22]. Thus, we monitored the expression level of Ctr1 in A2780 and A2780cisR cells. Data presented in Fig. 4a illustrated that Ctr1 is expressed at very low levels in A2780cisR cells in comparison to the Cisplatin sensitive A2780 cell line. This argues that reduced Ctr1 expression might contribute to the reduced sensitivity to Cisplatin observed in A2780cisR cells. To further evaluate the role of Ctr1 in Cisplatin resistance, we infected A2780 cells with three shRNA targeting the human Ctr1 gene. Data presented in Fig. 4b demonstrated that the expression of Ctr1 in the resulted clones, #352, #349, and #348, were silenced by 60, 5 and 90 %, respectively (Fig. 4b). Next, we evaluated the consequence of reduced expression of Ctr1 on Cisplatin uptake using ICP-MS. Figure 4c showed that uptake of Cisplatin into A2780 was significantly higher in comparison to A2780cisR. Moreover, silencing of Ctr1 in clone #348 caused a significant reduction (30 %) in Cisplatin uptake, but still higher than that of A2780cisR, arguing for the possibility that other transporters, alongside Ctr1, contribute to Cisplatin uptake in A2780 cell lines [23]. Next, we evaluated the uptake of RJY6 and RJY13 into the different A2780 cells. Figure 4d showed that uptake of RJY13 and RJY6 was efficient in all tested cells. Moreover, uptake of RJY13 and RJY6 was significantly higher than that of Cisplatin. For example, A2780 cells accumulated 0.00014 platinum /cells when they were exposed to 50 microM Cisplatin for 1 h. The same cells accumulated 0.0054 and 0.0041 platinum/cells when RJY6 and RJY13 were used, respectively, an increase of 30 fold in the uptake of the derivatives, which might explain, in part, the enhanced potency of RJY6 and RJY13 compared to the parental drug, Cisplatin. Furthermore, uptake of RJY11, a relatively non-active platinum (IV) derivative, was close to background in all A2780 cells, arguing that the activity of the different derivatives correlates with their cellular uptake (data not shown). Interestingly, uptake of RJY6 was significantly lower in A2780 cells with silenced Ctr1 (clone #348), and that in contrast to uptake of RJY13 that was not dependent on the presence of Ctr1 protein (Fig. 4d).

## Discussion

Previously, we evaluated the activity of a number of platinum (IV) prodrugs for their anti-proliferative and clonigenicity inhibition of the CML cell lines (Najajreh et al., in-preparation). In this study we focused on different parameters of our platinum (IV) series and evaluated anti-cancer activity targeting the A2780 and A2780cisR, Cisplatin sensitive and resistant ovarian cancer cell lines, respectively, including anti-proliferative, clonigenicity inhibition, apoptosis induction and drug uptake. In addition, we monitored the ability of our novel compounds to exert anti-proliferative and clonigenicity inhibition against inherently Cisplatin resistant cell lines such as HT29 and PC3 cells. In agreement with our previous data, two platinum (IV) prodrugs, RJY6 and RJY13, exhibited potent activity against ovarian cancer cells with comparable potency. In contrast to Cisplatin, our novel Cisplatin derivatives exhibited comparable activity against Cisplatin sensitive (A2780) and resistant (A2780cisR) ovarian cancer cells. However, we noticed a difference in the behavior of the two Cisplatin derivatives. While RJY13 exhibited strong anti-tumor activity in all in vitro assays, RJY6 was less potent in experiments that were performed in 2D conditions (anti-proliferation and apoptosis inducing assays) and exhibited strong activity in clonigenicity inhibition. Interestingly, we also observed significant differences in the selectivity profile between RJY6 and RJY13 against the non-cancerous cells, such as the HFF and HEK293A/T cells. In general, RJY 6 was more selective compared to RJY13. For example, by calculating the ratio of IC_50_s exhibited against none cancerous cells (HFF) to the cancerous cell line (A2780), we observed a ratio of 63, 30 and 117.2 when Cisplatin, RJY13, and RJY6, respectively were used. Moreover, comparable outcome were obtained when we compared the relative toxicity to HEK293A in relation to A2780 cells. This argues for an expected better toxicity profile of RJY6, while an enhanced toxicity of RJY13, in comparison to Cisplatin, when using *in vivo* systems. The mechanisms underlining the differences in behavior of the two compounds are not known and examining the in vivo toxicity is required to evaluate the full potential of our platinum (IV) prodrugs. In addition, mechanisms responsible for the reduced activity of RJY6 in 2D conditions in comparison to assays performed in 3D condition are yet to be determined. However, we hypothesized that differences might relate to varying exposure time in the two types of experiments. While 3D experiments required longer exposure time compared to 2D assays (2 weeks compared to 1–3 days) and therefore the difference in activity might be due to in-efficient bio-conversion of RJY6 compared to RJY13, a hypothesis awaiting experimental validation.

To shed a light on the mechanism responsible for increased potency of RJY6 and RJY13 in comparison to Cisplatin, we evaluated the role of copper transporter, Ctr1, in the uptake of Cisplatin in comparison to the two active Cisplatin (IV) prodrugs. Initially, we noticed that the Cisplatin resistant cells (A2780cisR) expressed very minimal amount of Ctr1 protein in comparison to sensitive cell lines (A2780), arguing for a potential role of Ctr1 in the acquired Cisplatin resistance observed in A2780cisR cells (Fig. 4). Next, we utilized shRNA approach to silence the Ctr1 gene in the sensitive A2780 cell line. We observed that uptake of Cisplatin was high in A2780 Ctr1^+^ cells, reduced by 70 % in resistant A2780cisR and by 30 % in A2780 Ctr1^−^ (clone #348) cells (Fig. 4c), arguing that Ctr1 protein is partially required for efficient Cisplatin uptake and other transporters besides Ctr1 required for efficient influx of paltinum compounds such as Ctr2 [24] and Organic Cation Transporters (Oct 1, 2, 3 and Oct 6) [25, 26]. Interestingly, uptake of RJY6 was marginally reduced upon Ctr1 silencing by 25 %; arguing that uptake of RJY6 might be partially dependent on the Ctr1 transporter. In contrast, uptake of RJY13 was not affected by the reduced Ctr1 expression. In fact, uptake of RJY13 was slightly higher in Ctr1 silenced cells. Our current data are in agreement with previous data reported by Ishida et al., 2002 and Pabla et al., 2009 demonstrating that knockdown of Ctr1 reduced Cisplatin uptake into yeast and mammalian cells and blocked Cisplatin-induced cell death [23, 27]. Moreover, Holzer et al. 2004 reported that Ctr1 controls the cellular accumulation of Cisplatin, Carboplatin, and Oxaliplatin at low concentrations, however, accumulation of Oxaliplatin is not dependent on Ctr1 at higher concentrations [22]. Our working hypothesis argues that uptake of RJY6 and RJY13 are largely Ctr1-independent and probably more efficient than that of Cisplatin and hence the enhanced activity. Moreover, similarly to other reported platinum (IV) prodrugs, enhanced activity and ability to overcome chemo resistance might be related to the lipophilicity of the prodrugs that favors its cellular accumulation by passive diffusion [28]. We expected that our platinum (IV) prodrugs will exhibit similar cellular activity as platinum (II). However, this assumption needed to be experimentally validated. In some cases, Cisplatin prodrugs such as Oxoplatin exhibit different intracellular effects mediated by differences in induction of stress responses [29]. Nevertheless, our data illustrated the therapeutic potential of platinum (IV) prodrugs in overcoming Cisplatin chemo resistance in cancer cells and potential better in vivo cytotoxic profile for some of them.

## Conclusion

In this report we explored the therapeutic potential of our platinum (IV) prodrugs in inducing anti-cancer activity to Cisplatin resistant and sensitive ovarian cancer cell lines and showed that the uptake of Cisplatin is partially dependent on Ctr1 transporter, while uptake of our platinum (IV) prodrugs is largely Ctr1-independent. Moreover, our results demonstrated the potential of platinum (IV) prodrugs in overcoming acquired and inherited drug resistance in cancer cell lines and their therapeutic potential in cancer therapy.

## Abbreviations

2D: two dimensional
3D: three dimensional
A2780: cisplatin sensitive ovarian cancer cell line
A2780cisR: cisplatin resistant ovarian cancer cell line
Ad5: human species C adenovirus serotype 5
Akt: protein kinase B
CDDP: cis-Diamminedichloroplatinum (II) [*cis*-[PtCl_2_(NH_3_)_2_]
CML: chronic myelogenous leukemia
Ctr1: copper transporter-1
CV: coefficient of variation
DMEM: Dulbecco’s Modified Eagle’s Medium
DMSO: dimethyl sulfoxide
ELISA: enzyme-linked immunosorbent assay
ESIMS: electrospray ionization mass spectrometry
FBS: fetal bovine serum
FT-IR: fourier transform infrared spectroscopy
HEK293: human embryonic kidney cell
HFF: human Foreskin Fibroblast
HT29: colon cancer cell line
ICP-MS: inductively coupled plasma mass spectrometry
mTOR: mammalian target of rapamycin
MTT: 3-(4,5-dimethylthiazol-2-yl)-2,5-diphenyltetrazolium bromide
NMR: proton nuclear magnetic resonance
PARP: poly ADP ribose polymerase
PBS: phosphate-buffered saline
PMSF: phenylmethylsulfonyl fluoride
RPMI: Roswell Park Memorial Institute
SDS: sodium dodecyl sulfate
SV40: simian vacuolating virus 40
XTT: 2,3-bis-(2-methoxy-4-nitro-5-sulfophenyl)-2H-tetrazolium-5-carboxanilide
